# Assessment of significant coronary artery stenosis using blood oxygen level dependent cardiovascular magnetic resonance (BOLD-CMR)

**DOI:** 10.1186/1532-429X-14-S1-P4

**Published:** 2012-02-01

**Authors:** Jodi Harker, Judy Luu, James Hare, Dominik P Guensch, Matthias G Friedrich

**Affiliations:** 1 Stephenson CMR Centre, Libin Cardiovascular Institute, University of Calgary, Calgary, AB, Canada; 2 Baker IDI Heart & Diabetes Institute, Melbourne, VIC, Australia

## Summary

Oxygenation-sensitive CMR at 1.5T can be used to identify functionally significant coronary artery stenosis, by demonstrating a blunted response to adenosine induced hyperaemia. Image quality remains a limitation.

## Background

Using the magnetic properties of hemoglobin, changes in myocardial tissue oxygenation can be detected with blood oxygen level dependent (BOLD) cardiovascular MRI (CMR). The study aim was to assess whether BOLD-CMR images can detect an abnormal myocardial tissue response to adenosine infusion in patients with CAD, when compared to fractional flow reserve (FFR).

## Methods

Patients undergoing clinically indicated coronary angiography underwent BOLD CMR scans using a clinical 1.5T scanner. Three short axis BOLD cine images were captured at baseline and during adenosine-induced coronary hyperemia. The mean segmental percent signal intensity (SI) changes were calculated between baseline and hyperemia in the subendocardial myocardium using the 16-segment model. Segments were defined as ischemic or non-ischemic by FFR (cut-off <0.80). The segment with the lowest BOLD SI percent change per patient was used for analysis.

## Results

Thirty-two patients were enrolled, 5 patients were excluded due to incomplete CMR, leaving 27 patients (age 61 ± 10 years) for analysis. There were 864 myocardial segments (baseline and adenosine) available for analysis, 289 were subtended by a coronary artery with an available FFR value. Eighty-two segments (28%) were excluded due to pre-defined criteria for poor image quality, 67% were apical. From the remaining 20 patients, 7 had ischemic FFR values and 13 had non ischemic FFR values. Using the segment with the lowest % BOLD SI change per patient there was a significant difference between ischemic -6.49% ± -8.65% and non ischemic 4.21 ± 4.94% (p=0.0023) patients. Using a cut off value of 1.1% SI change the sensitivity is 86%, specificity 69%, positive predictive value 0.6 and negative predictive value 0.9.

## Conclusions

A blunted hyperemic response to adenosine as assessed by oxygenation-sensitive CMR at 1.5T can identify functionally significant coronary artery stenosis. However, image quality, mainly in apical segments, remains a limitation.

## Funding

Canadian Institute of Health Research.

**Figure 1:**
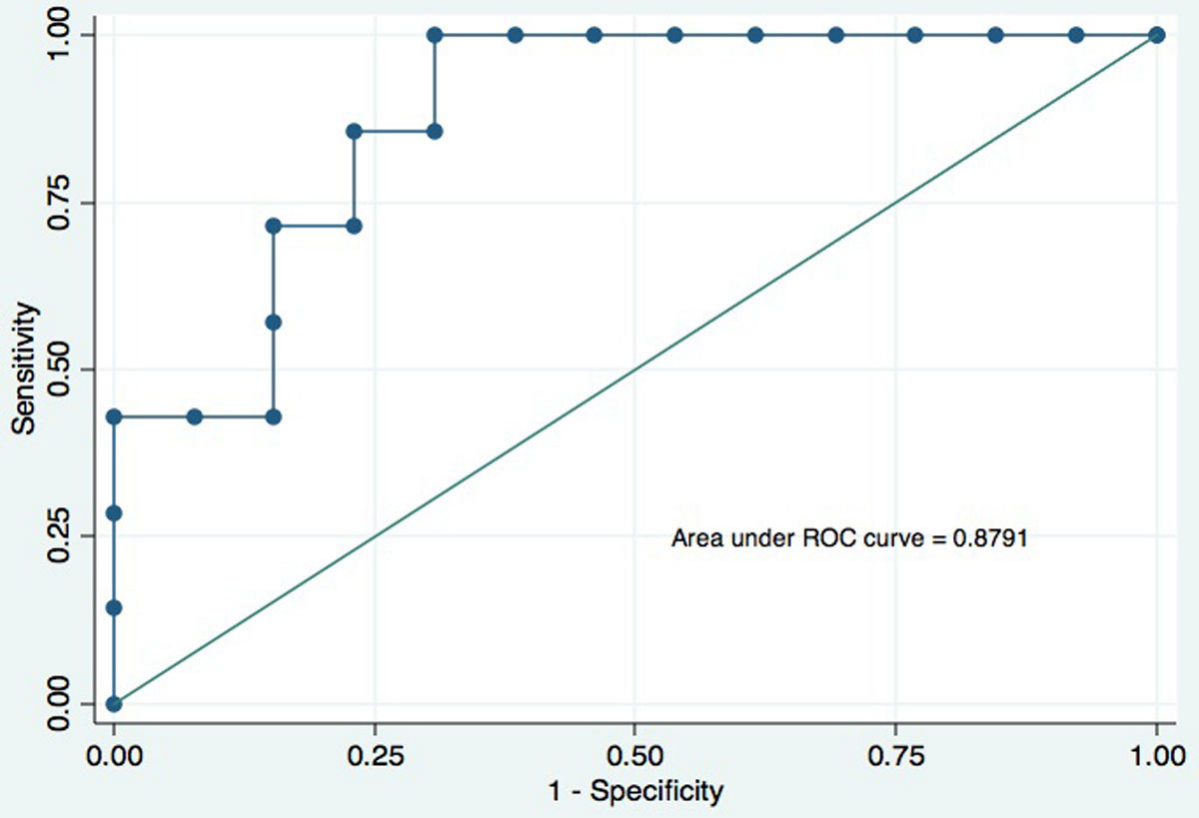
Receiver operating characteristic (ROC) curve for BOLD %SI change.

